# Unraveling progression subtypes in people with Huntington’s disease

**DOI:** 10.1007/s13167-024-00368-2

**Published:** 2024-05-28

**Authors:** Tamara Raschka, Zexin Li, Heiko Gaßner, Zacharias Kohl, Jelena Jukic, Franz Marxreiter, Holger Fröhlich

**Affiliations:** 1 Department of Bioinformatics, Fraunhofer Institute for Algorithms and Scientific Computing (SCAI), Schloss Birlinghoven, 53757 Sankt Augustin, Germany; 2 Bonn-Aachen International Center for IT, University of Bonn, Friedrich-Hirzebruch-Allee 6, 53115 Bonn, Germany; 3 Department of Molecular Neurology, University Hospital Erlangen, Friedrich-Alexander-Universität Erlangen-Nürnberg, 91054 Erlangen, Germany; 4 Fraunhofer IIS, Fraunhofer Institute for Integrated Circuits IIS, Am Wolfsmantel 33, 91058 Erlangen, Germany; 5 Department of Neurology, University of Regensburg, Regensburg, Germany; 6 Center for Movement Disorders, Passauer Wolf, 93333 Bad Gögging, Germany; 7 Center for Rare Diseases Erlangen (ZSEER), University Hospital Erlangen, Friedrich-Alexander-Universität Erlangen-Nürnberg, 91054 Erlangen, Germany

**Keywords:** Huntington’s disease, Progression, Artificial intelligence, Cognition, Non-motor symptoms, Predictive preventive personalized medicine, Patient stratification, Precision medicine

## Abstract

**Background:**

Huntington’s disease (HD) is a progressive neurodegenerative disease caused by a CAG trinucleotide expansion in the huntingtin gene. The length of the CAG repeat is inversely correlated with disease onset. HD is characterized by hyperkinetic movement disorder, psychiatric symptoms, and cognitive deficits, which greatly impact patient’s quality of life. Despite this clear genetic course, high variability of HD patients’ symptoms can be observed. Current clinical diagnosis of HD solely relies on the presence of motor signs, disregarding the other important aspects of the disease. By incorporating a broader approach that encompasses motor as well as non-motor aspects of HD, predictive, preventive, and personalized (3P) medicine can enhance diagnostic accuracy and improve patient care.

**Methods:**

Multisymptom disease trajectories of HD patients collected from the Enroll-HD study were first aligned on a common disease timescale to account for heterogeneity in disease symptom onset and diagnosis. Following this, the aligned disease trajectories were clustered using the previously published Variational Deep Embedding with Recurrence (VaDER) algorithm and resulting progression subtypes were clinically characterized. Lastly, an AI/ML model was learned to predict the progression subtype from only first visit data or with data from additional follow-up visits.

**Results:**

Results demonstrate two distinct subtypes, one large cluster (*n* = 7122) showing a relative stable disease progression and a second, smaller cluster (*n* = 411) showing a dramatically more progressive disease trajectory. Clinical characterization of the two subtypes correlates with CAG repeat length, as well as several neurobehavioral, psychiatric, and cognitive scores. In fact, cognitive impairment was found to be the major difference between the two subtypes. Additionally, a prognostic model shows the ability to predict HD subtypes from patients’ first visit only.

**Conclusion:**

In summary, this study aims towards the paradigm shift from reactive to preventive and personalized medicine by showing that non-motor symptoms are of vital importance for predicting and categorizing each patients’ disease progression pattern, as cognitive decline is oftentimes more reflective of HD progression than its motor aspects. Considering these aspects while counseling and therapy definition will personalize each individuals’ treatment. The ability to provide patients with an objective assessment of their disease progression and thus a perspective for their life with HD is the key to improving their quality of life. By conducting additional analysis on biological data from both subtypes, it is possible to gain a deeper understanding of these subtypes and uncover the underlying biological factors of the disease. This greatly aligns with the goal of shifting towards 3P medicine.

**Supplementary Information:**

The online version contains supplementary material available at 10.1007/s13167-024-00368-2.

## Introduction

### The role of CAG repeat length in predicting disease onset

Huntington*’*s disease (HD) is a progressive, autosomal-dominantly inherited neurodegenerative disease, caused by a CAG trinucleotide expansion in the huntingtin gene [1]. CAG repeat lengths > 39 show full penetrance, and length of the CAG repeat is inversely correlated with disease onset [2, 3]. Core feature of HD is a hyperkinetic movement disorder, called chorea. In addition, psychiatric symptoms and cognitive deficits are an essential part [4–6], predominantly influencing patient’s quality of life [7–10], and are often early signs of HD [4, 11]. This gives great potential for predictive, preventive, and personalized medicine (3PM) to improve diagnostic and treatment of patients.

The “CAG age product” (CAP) that is, as the name intends, based on age and CAG repeat length can predict the age of onset of the disease [12, 13]. The CAP is commonly collected and used in clinical studies to determine the pre-manifest status of the patients or to assess the disease stage in terms of being close to or far away from predicted onset at study entry [12, 14–16]. Nevertheless, although CAG repeat length is often measured in a predictive manner in HD patient’s relatives, no preventive actions are taken, as no preventive treatment is available.

Taking into account the genetic component and the clear psychiatric symptoms and cognitive deficits in HD patients, there is great potential for the aspects of 3P medicine, as the clinical diagnosis of manifest HD is only based on the presence of unequivocal motor signs in CAG repeat expansion carriers and ignores the other aspects [17–19].

### The need for a 3PM approach

Although there is such a clear genetic background in HD, the multifactorial and progressive nature of HD, in combination with a long pre-manifest phase again strengthens the need for 3PM concepts in HD, as there is a high variability of symptoms in HD patients that challenges counseling [6, 20, 21].

Commonly, clinical diagnostic criteria feature a pre-manifest phase (prior to motor diagnosis), followed by the motor-manifest period, divided into five stages [17]. More recently, a biological classification system has been established to characterize individuals for research purposes [22] and longitudinal data-driven machine-learning algorithms identified even nine distinct disease stages [23].

These results have tremendously helped to better understand the natural course of HD on a population level and paves the way for significantly earlier and correct diagnosis, enabling the paradigm shift from delayed intervention towards predictive and personalized medicine. Earlier diagnosis in the end helps to identify and apply effective early interventions for HD patients in early disease stages [19, 24, 25].

Therefore, the urgent need for personalized medicine has to be addressed. Here, identifying patient subtypes with a similar disease trajectory could help to untangle disease-modifying factors and define patient stratification [21, 24, 26–29]. Such an approach aims, on the one hand, to identify the best treatment strategy for each individual disease subtype, but, on the other hand, also helps to understand the disease characteristics itself, which in the end will contribute to a better prediction of disease progression, as well [24, 29].

### State of the art in subtype identification in neurodegenerative diseases

Several models have been developed that cluster and predict patient subtypes based on their longitudinal trajectories, but most of them do not account for variations in the dynamics of the disease progression or identified markers discriminating pre-defined progression groups [21, 23]. In fact, a fully multivariate data-driven approach to unravel potential underlying disease progression groups, that otherwise can be overseen, is still missing. In this regard, methods from the field of data science and artificial intelligence (AI) pose a great opportunity to come closer to the vision of a predictive, personalized, and preventive medicine (3PM).

In the past, data-driven clustering of multisymptom disease trajectories has shown promising results in other neurodegenerative disorders, such as Alzheimer’s or Parkinson’s [28, 30, 31]. The rate of progression is typically variable across the disease trajectory in HD, with the steepest decline in function being seen in early to mid-disease stages [32]. Also, patients may be initially diagnosed at different stages of their disease. Thus, there is a need for alignment of trajectories observed for an individual patient along a population average disease trajectory [33, 34]. This can be done by modeling a continuous common disease timescale, e.g., with the help of non-linear mixed effect models. These models enable a removal of the effect of the actual time point of symptom onset on the overall trajectories by assessing a latent time estimate and adjust all patients’ trajectories across a common disease timescale [33, 35, 36].

### Novelty beyond the state of the art

After diagnosis, accurate and early prognosis of the progression of the disease is of core relevance for those living with HD. For a patient, it is important to know her prospective symptom development in order to adapt life accordingly. In addition, a concrete prognosis could help doctors to better organize and manage therapies. This is where our work contributes to. For the first time, to our knowledge, HD subtypes based on multisymptom disease trajectories were identified and validated. Here, not only motor symptoms were evaluated but also cognitive symptoms are taken into account for subtype identification, as psychiatric symptoms and cognitive deficit are an essential part of the disease and also early signs of HD that are ignored in the diagnosis of manifest HD [4–6, 11, 17–19].

With the help of an AI/ML model that allows for predicting the disease progression subtypes based on only the first visit or by including additional follow-up visits, we addressed important 3PM aspects, because such a model could support a better individualized disease management via optimized counseling as well as support patient in their own life decisions.

### Working hypothesis

In the current study, we hypothesize that manifest HD patients define heterogeneous progression subtypes that can be identified by advanced AI methods. Subtype identification will allow the classification of patients into one of the multisymptom trajectory clusters based on their first visit data only or by including additional follow-up visits. This will in the end result into a better personalized and preventive treatment for HD patients as patient counseling can be optimized. Additionally, patients can be provided with a better indication of their prognosis.

Therefore, we clustered HD patients on the basis of their longitudinal trajectories using our previously introduced Variational Deep Embedding with Recurrence (VaDER) neural network algorithm [30]. As such trajectories are often temporally related to the study baseline, this study aims to adjust for this confounding effect using a non-linear mixed effect model, originally introduced by Raket [36], which assumes that the longitudinal trajectories of pre-manifest and manifest HD patients are adjustable along a common disease timescale. An early prognosis of disease progression for each patient, reflecting the predictive and personalized aspect of 3PM, will be achieved by developing, evaluating, and validating a machine learning-based prediction model.

## Methods

### Dataset and patient selection criteria

Data used in this work were generously provided by the participants in the Enroll-HD study [37] (ClinicalTrials.gov Identifier: NCT01574053) and made available by CHDI Foundation, Inc. Enroll-HD is a global clinical research platform designed to facilitate clinical research in Huntington’s disease. Core datasets are collected annually from all research participants as part of this multicenter longitudinal observational study. Data are monitored for quality and accuracy using a risk-based monitoring approach. All sites are required to obtain and maintain local ethical approval. Overall, up to now, over 20,000 patients from 21 countries at 159 sites were recruited [38]. Enroll-HD provides data from HD patients, including manifest and pre-manifest patients. The study has collected clinical score data and demographics during annual visits. During these visits, patients undergo a core battery of test, including demographics, medical history, and clinical assessments of the four HD domains: motor, cognitive, behavioral, and functional. A CAG genotyping was done for each participant to get their CAG repeat length.

During this study, we used the fifth release of the Enroll-HD data from October 2020 that was released in December 2020. Within this release, we selected the information from scheduled visits of manifest and pre-manifest patients that had at least one follow-up visit after the baseline screening. Control participants were excluded for the purpose of this study. Participant category was assessed at each visit by the study examiner. Details about the definition of pre-manifest and manifest patient group can be found in the Supplementary Material.

Used features during modeling and clustering of trajectories are the Unified Huntington’s Disease Rating Scale (UHDRS) [39] and the mini-mental state examination (MMSE). The UHDRS consists of four different domains: motor, cognitive, behavioral, and functional, where each of the domains relates to different fields of possible symptoms. Additionally, the MMSE was used as a cognitive test. In total, we used three different features, coming from two different assessments, namely, UHDRS total motor score (TMS), UHDRS symbol digit modality test (SDMT), and the total MMSE score (MMSE). Furthermore, age and sex were used as covariates. Characteristics of all used patients can be found in Table 1.

**Table 1:** Characteristics of manifest and pre-manifest patients

	Manifest	Pre-manifest
Number of patients	$7533$	$372$
Age	$52.9\pm 12.53$	$47.01\pm 12.06$
Sex		
Male	$3659$	$170$
Female	$3874$	$202$
CAG length	$43.96\pm 3.86$	$43.3\pm 3.32$
UHDRS		
Total motor score	$36.92\pm 21.07$	$14.19\pm 8.44$
Symbol digit modality test	$23.39\pm 13.06$	$38.95\pm 12.76$
MMSE	$25.00\pm 4.39$	$28.01\pm 2.12$

Shown are statistics of patients used for the subtype analysis as training (manifest) and validation (pre-manifest) set. Statistics are shown at patients’ baseline visit for manifest and first manifest visit for pre-manifest patients after conversion. The age, CAG repeat length, and clinical scores are described by their mean and standard deviation.

### Model building methods

In the following analysis, both manifest and pre-manifest patients were used for modeling common disease time trajectories with a non-linear mixed effect (NLME) model. VaDER clustering and prediction models were then trained on manifest patients only, while validating these models afterwards on pre-manifest patients. Common disease timescale trajectories of pre-manifest patients were therefore shortened to their manifestation phase, meaning that the first visit when manifesting the disease was considered the start of their used trajectory.

#### Non-linear mixed effect model

The here-used model follows the rational described earlier [33, 36]. Shortly, it is a non-linear mixed effect model with a mean curve defined by the fixed effects and random effects describing the deviation of each patient from that mean curve. During the here-described analysis, the mean curve is formulated as a generalized logistic function:

$$\mu \left(t\right)=A+\frac{K-A}{\left(1+{e}^{{\left(-B\left(t+s\right)\right)}^{v}}\right)}+c$$

where $A$ is the left and $K$ the right asymptotic value which reflects the minimal and maximal possible value of a specific clinical measure. Parameters $B$ and $v$ define the curvature of the function, with $B$ as time scaling parameter and $v$ as an asymmetry parameter. A shift in time is modelled by $s$ as horizontal shift and a vertical shift can be modelled by $c$.

During the modeling, the values of $A$ and $K$ are fixed according to the actual modelled clinical score. Fixed effects can be modelled for specific covariates, e.g., the manifestation status of the patients at baseline, such that the parameter estimate of $s$ describes the mean difference in time between the pre-manifest and manifest patient groups over the continuous common disease timescale. This difference is modelled relative to the pre-manifest patient group, meaning that $t=0$ on the common disease timescale corresponds to the average status of the pre-manifest patients at the time of conversion into manifest disease status.

The model was built with the help of the progmod R-package [40] which is based on the nlme package [41]. More details on the model formulation can be found in the Supplements.

#### Multivariate clustering of clinical trajectories

The previously published Variational Deep Embedding with Recurrence (VaDER) [30] was used to cluster the shifted time series that are the output of the nlme model. Based on a variational deep embedding framework for learning low-dimensional representation of data points, two long short-term memory networks that model the multivariate time series, and an implicit imputation layer, the VaDER method allows to model and cluster short time series with large amount of missingness. More details can be found in the original publication [30] and in the Supplements. Training of the VaDER was based on manifest patients only, but pre-manifest patients were used as validation cohort in the later phase of the study.

#### Machine learning classifiers

Random Forest [42] and XGBoost [43] classifiers were trained on baseline data only (BL), as well as baseline and follow-up data (BLtoFU1 and BLtoFU2) from manifest patients. We here used the labels of the VaDER approach, the cluster assignment, as the classes that need to be predicted. As predictors, multiple cognitive, motor, functional, and neurobehavioral scores were used. Those can be found in Supplementary Table 3. A hyperparameter optimization with randomized search with 100 parameter settings preceded a tenfold nested cross-validation implemented with scikit-learn [44] and XGBoost [43] packages. Hyperparameter spaces and optimal hyperparameters, found based on the best AU-ROC, can be found in the Supplementary Material. Cross-validation results of both Random Forest and XGBoost are shown in the Supplementary Material, along with the decision to choose XGBoost for final modeling due to higher prediction performance. These trained XGBoost models were then applied on the pre-manifest patients as a validation set in the later phase of the study.

### Feature importance analysis

For feature importance analysis, SHAP values [45] for each feature in the model were calculated with the implementation of the python package *shap*. For interpretation, the natural additive behavior of SHAP values was used to calculate aggregated SHAP values for specific domains of clinical tests, such as functional, cognitive, or neurobehavioral tests. This was done by summarizing SHAP values from clinical tests belonging to each of the domains. The scores belonging to each of the domains are listed in Supplementary Table S3.

### Statistical testing

Statistical testing was following the SHAP analysis for all single features underlying the top aggregated features. Depending on the type of variable, either Kruskal–Wallis (numerical), Fisher’s exact (bi-categorical), or chi-square independence test (multicategorical) was used to test the distribution of the respective features within the found subtypes. Age and sex were included as possible confounders in all tests and *p*-values were corrected for multiple testing using the Benjamini–Hochberg procedure.

The analysis of medications for specific indications across progression subtypes was performed using Fisher’s exact test, and *p*-values were corrected for multiple testing using Benjamini–Hochberg procedure.

## Results

### Data

Data used in this study comes from the Enroll-HD study (ClinicalTrials.gov Identifier: NCT01574053) [37] (Data Cut 10/2020). In total, 11,093 patients (7548 manifest and 3545 pre-manifest) were used in this work for the longitudinal modeling over a common disease timescale, of which 7905 patients (7533 manifest and 372 pre-manifest) were eligible for the later analysis regarding the subtypes. For pre-manifest patients, only their manifest phase was used in the later analysis as independent validation set, explaining the large drop in the number of patients. An overview of the characteristics of patients used for the subtype analysis at their first included visit is presented in Table 1.

Enroll-HD is a longitudinal study, collecting data from patients at multiple annually scheduled follow-up visits. The number of available patients per follow-up visit is shown in Table 2.

**Table 2:** Number of baseline and follow-up visits

	BL	FU1	FU2	FU3	FU4	FU5	FU6	> 6FUs
Manifest	$7533$	$7066$	$4817$	$3256$	$1922$	$774$	$211$	$62$
Pre-manifest	$372$	$372$	$213$	$94$	$34$	$9$	*-*	*-*

Shown is the number of manifest and pre-manifest patients used in the subtype analysis having the respective visits available; e.g., 7066 manifest patients had a first follow-up visit, while 4817 manifest patients also had a second follow-up visit. For manifest patients, available visits over 6 years were summarized for visual reasons. The maximum number of visits a manifest patient had was 14. Baseline visit for pre-manifest patients relates to their first manifest visit, after conversion from pre-manifest status.

### Non-linear mixed effect model

**Fig. 1:**
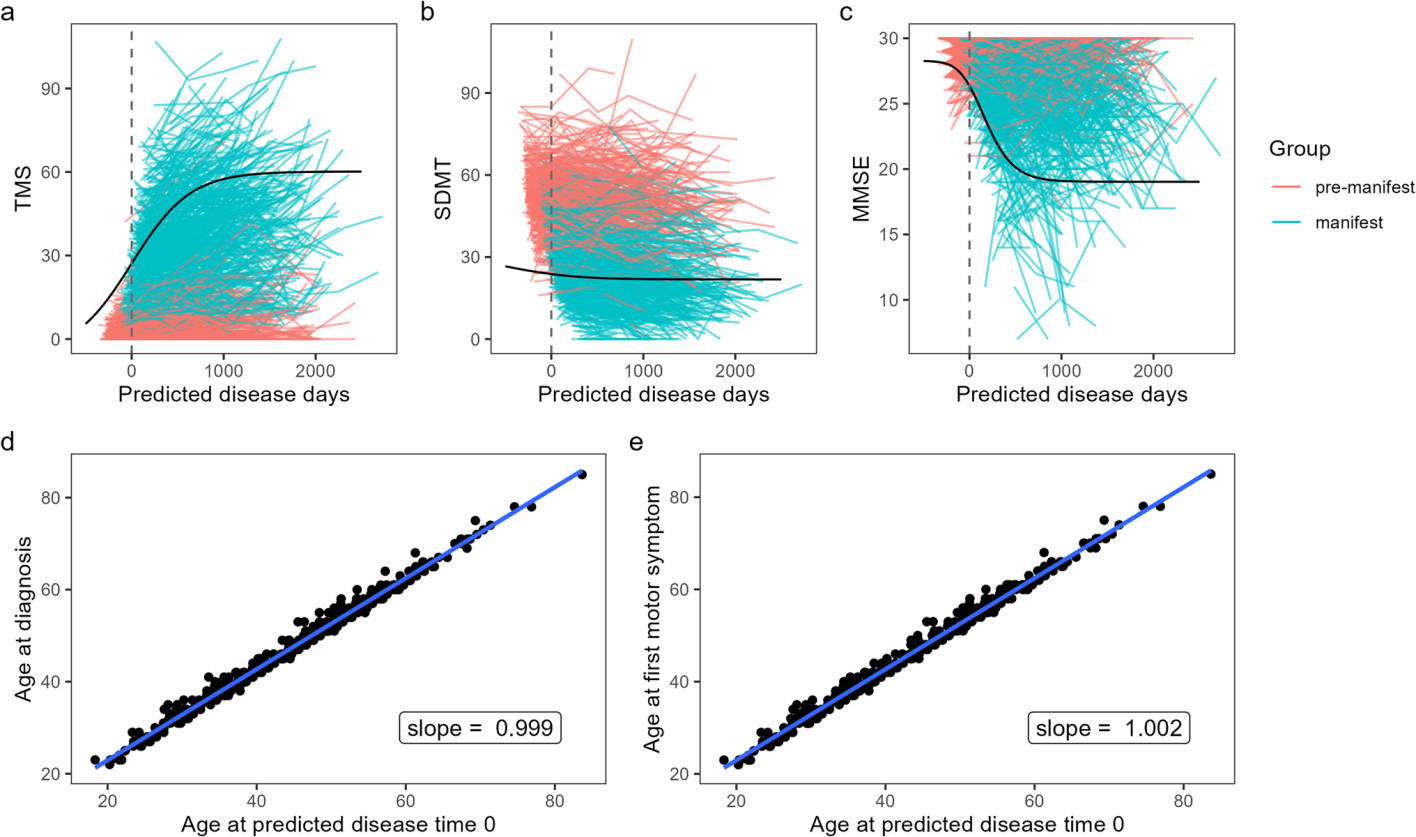
Multivariate NLME modeling. **a**–**c** Disease trajectories of pre-manifest (red) and manifest (blue) patients aligned along a common disease timescale based on the estimated random effects representing the latent time. Three different outcomes are shown: Unified Huntington’s Disease Rating Scale (UHDRS) total motor score (TMS), symbol digit modality test (SDMT), and mini-mental state examination (MMSE). For visualization reasons, 1000 trajectories were randomly selected for plotting. The black curve shows the underlying mean curve estimated by the NLME model. The grey dashed line marks time equals 0 on the common disease timescale. **d**, **e** Validation plots of time alignment, where age at diagnosis (**d**) and first motor symptom (**e**) are plotted against the age at predicted disease time 0 fitted with linear models resulting from the NLME approach

A non-linear mixed effect (NLME) model was fitted to longitudinal data of 11,093 manifest and pre-manifest patients in a multivariate manner, modeling the Unified Huntington’s Disease Rating Scale (UHDRS) total motor score (TMS), UHDRS symbol digit modality test (SDMT), and mini-mental state examination (MMSE). These scores represent current gold standard scores addressing motor and cognitive aspects of the disease [46, 47]. The NLME model gives the opportunity to align trajectories on a common, potentially unobserved (i.e. latent), disease timescale. Figure 1a–c shows the aligned trajectories of pre-manifest and manifest patients of the three modeled clinical scores along the common disease timescale. As expected, the original trajectories of pre-manifest patients are mostly shifted towards the left (back in time). Vice versa, manifest patients’ trajectories are mostly shifted to the right, as patients naturally are in pre-manifest state before their disease manifests. Resulting latent time estimates, indicating the difference between the actual time axis and the common disease timescale, are validated by correlating the predicted age at time 0 (i.e. manifestation time) for each individual against the observed age at first diagnosis and first motor symptoms. Hereby, a linear regression with a slope of 1 would indicate that observed diagnosis and predicted symptom onset are consistent and therefore, trajectories would be perfectly aligned. Results (Fig. 1d, e) in this study show slopes of 0.999 (diagnosis) and 1.002 (first motor symptoms), demonstrating a good fit of the NLME model to the observed data. Hence, estimates of latent time seem reliable.

### VaDER clustering results in two subtypes

**Fig. 2:**
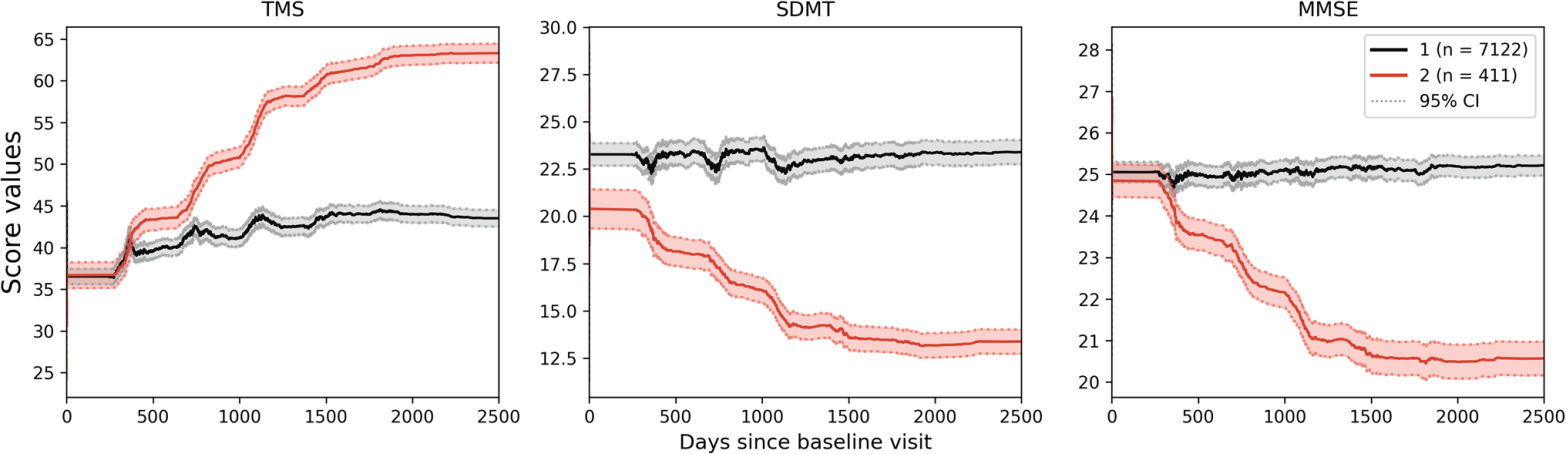
Mean progression cluster trajectories of manifest HD patients. Two clusters are found as the result of training VaDER on the aligned multisymptom (TMS, SDMT, MMSE) trajectories of manifest HD patients. Cluster 1 is shown in black and contains 7122 patients, whereas Cluster 2 (red) only contains 411 patients. Dashed lines indicate the 95% confidence interval of the mean trajectories. Overall, the first cluster shows more stable patterns over time compared to the second cluster

Aligned trajectories of 7533 manifest HD patients were used to train a VaDER model for clustering patients into subtypes [30]. This resulted in two clusters, of which one cluster was a large one with 7122 patients included, while the second cluster only contained 411 patients (Fig. 2). While the second cluster shows a steep decline of SDMT and MMSE and rise of TMS starting from around day 200 on the common disease timescale, the first cluster demonstrates almost no impairment of patients over all three outcome scores and the whole common disease timescale, meaning that the large cluster and thus most HD patients show a stable pattern in the clinical scores after a minimal worsening in the beginning of their disease. However, a smaller subset of particularly vulnerable patients represents faster disease progression than most HD patients. Additionally, SDMT progression patterns of the two clusters are strictly distinguished of each other. Here, the baseline levels are already different, whereas both clusters are not separable in the beginning when looking at the MMSE or TMS.

### Predicting the HD progression subtype from clinical data

**Fig. 3:**
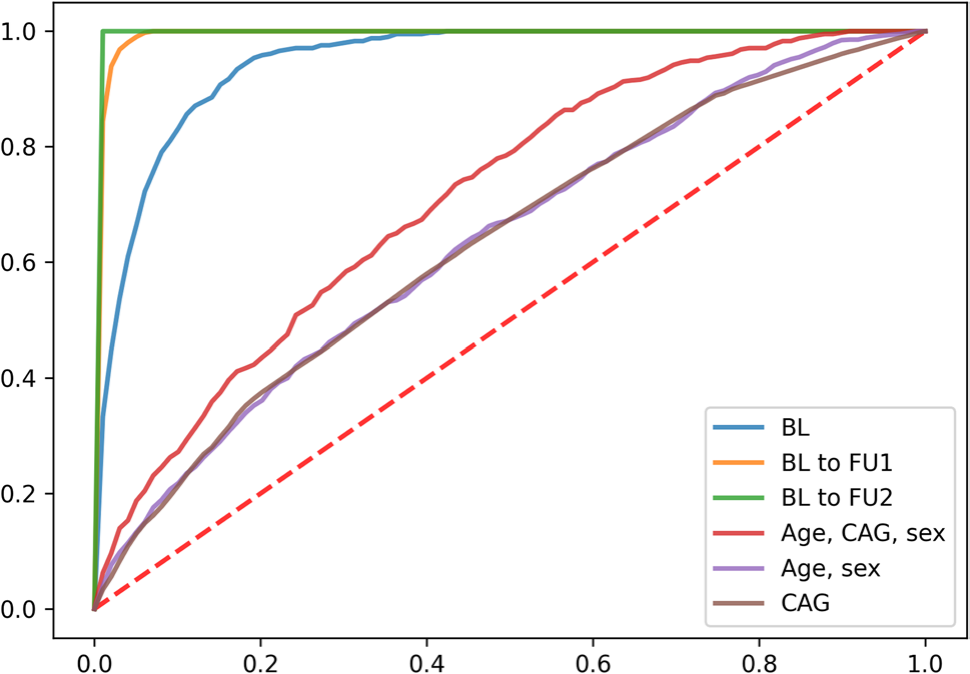
XGBoost classifier ROC curves. Mean ROC curves from tenfold cross-validation setting, are shown for multiple cases of prediction from clinical data, based on baseline visit only (BL, blue), baseline plus first follow-up visit (BLtoFU1, orange), or baseline plus first and second follow-up visit (BLtoFU2, green). The training performance of the three classifiers is summarized via the area under the ROC curve (AUC) of the mean ROC curve and is 95%, 99%, and 99%, respectively. Additionally, ROC curves from ground-truth classifiers based on only age, sex, and CAG repeat (red); age and sex (purple); or only CAG repeat (brown) are shown. Here, AUCs are 71%, 64%, and 63%)

We trained an XGBoost classifier to predict the progression subtype of a patient only using baseline data (BL), or additional data from the next (BLtoFU1) or next two visits (BLtoFU2). We used multiple cognitive, motor, functional, and neurobehavioral scores, as well as demographic and medical history features, as predictors. Those can be found in Supplementary Table S3. All classifiers were trained in a tenfold cross-validation scheme. That means we systematically and sequentially held 10% of the patients out for testing our classifier, while the training was performed on the rest of the data. Resulting receiver operator characteristic (ROC) curves are shown in Fig. 3 and indicate a high area under ROC curve (AUC) of 95% (BL), 99% (BLtoFU1), and 99% (BLtoFU2), respectively. This shows that classification performance grows with including additional follow-up visits, although the accuracy based on only the baseline visit is already high. Hence, first visit data alone already contains sufficient signal to make an accurate prognosis regarding subsequent progression pattern of the disease. A comparison to ground-truth models, containing either only CAG repeat, age plus sex, or all three confounders together, shows that the models using additional clinical data are significantly better (CAG 63%; age and sex 64%; age, sex, and CAG 71%).

#### Feature importance analysis

**Fig. 4:**
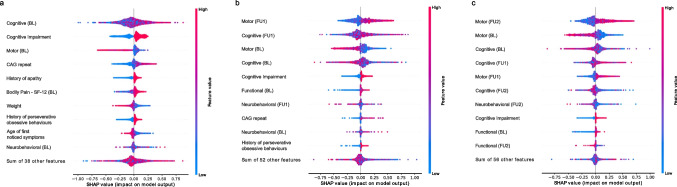
SHAP values of TOP10 aggregated features. The impact of the features based on aggregated SHAP values is shown here for BL (**a**), BLtoFU1 (**b**), and BLtoFU2 (**c**) XGBoost classifier. Multiple features were aggregated for the SHAP analysis based on the same domain of clinical tests, namely, cognitive, motor, functional, and neurobehavioral tests. A higher positive SHAP value indicates a higher influence of a feature to predict a patient as fast progressing. A more negative value indicates a higher tendency towards predicting the patient as slow progressing. The actual value of the feature is shown in a color code. The darker the red color, the higher the feature value

We conducted an analysis using Shapley Additive Explanations (SHAP) to better understand the contribution of individual features in the machine learning classifiers. Here, multiple motor, cognitive, functional, and neurobehavioral scores were aggregated within their domains. A complete list of which scores were included in each domain can be found in Supplementary Table S3. The SHAP analysis demonstrated that cognitive scores were very important for predicting the correct HD progression subtype in all classifiers, most influencing in the BL classifier (Fig. 4). However, also motor scores played a relevant role, specifically in the BLtoFU2 classifier, which employs information of the second follow-up visit. Additionally, the number of CAG repeats was among the most influential features for the BL and BLtoFU1 classifiers. Moreover, neurobehavioral scores had a strong impact on all models. The amount of cognitive impairment, observable apathy, and a history of perseverative obsessive behaviors were among the top important features in the BL classifier, but were becoming less influencing when including more follow-up visits. Interestingly, functional scores became more important for the subtype prediction when including more follow-up visits.

#### Clinical characterization of progression subtypes

In addition to the SHAP analysis, statistical tests regarding the differences in the features, aggregated in the top 10 most important features for the prediction of progression subtypes, were conducted (see details in the “Methods” section). All tests were adjusted for age and sex as possible confounders and corrected for multiple testing. For the BL classifier, among the five most significantly different single features were the cognitive impairment ($p<$ 3.3e^−13^) and multiple cognitive assessments, like categorical verbal fluency ($p<$ 3.3e^−14^), letter verbal fluency ($p<$ 3.3e^ −14^), and Trail Making Test Part A ($p<$ 3.4e^−9^) and B ($p<$ 4.5e^−16^). The amount of patients with cognitive impairment is higher in the second, smaller cluster, which is in concordance with the result, that the time needed for completion of Trail Making Test Part A and B is higher, and the number of correct answers in the categorical and letter verbal fluency tests is lower in this cluster than in the first, large cluster. Visualization of the distribution of these features can be found in the Supplementary Material. In the case of BLtoFU1 and BLtoFU2 classifiers, cognitive assessments were among the top significantly different features as well. Here, the SDMT (BLtoFU1: $p<$ 1.4e^−32^; BLtoFU2: $p<$ 2.1e^−35^ (FU2)) and Stroop tests (color naming: $p<$ 1.6e^−23^ (BLtoFU1), $p<$ 4.4e^−31^ (BLtoFU2); inference: $p<$ 5.7e^−^^23^ (BLtoFU1)) are present. The number of correct answers in the Stroop and SDMT is lower in the second cluster, as well. Also, the MMSE scores are significantly (BLtoFU1: $p<$ 3.0e^−^^30^; BLtoFU2: $p<$ 1.2e^−^^30^) lower in the second cluster. Therefore, subtypes were mostly distinguished by the amount of cognitive impairment of patients at baseline. Additionally, the TMS is significantly higher (BLtoFU1: $p<$ 3.8e^−^^26^, BLtoFU2: $p<$ 1.1e^−^^48^) in the second cluster. Furthermore, CAG repeat length, which is known to be a highly predictive feature of HD onset [3, 48], was also a predictive feature for subtype assignment and thus for HD progression. A complete list of the statistical test results can be found in Supplementary Table S7.

With respect to medication, intake of medication for five different indications, particularly relevant to HD, was compared between subtypes: chorea, depression, anxiety, irritability, and cognitive decline. Here, significant differences could be observed across the progression subtypes for chorea ($p=$ 0.0002), irritability ($p=$ 0.0037), and anxiety ($p=$ 0.0079) and while no significant difference between the progression groups were found for depression ($p=$ 0.0892) or cognitive disorder ($p=$ 0.0981) medication. Distributions of each medication in each subtype can be found in Supplementary Fig. S3.

### Application of model on pre-manifest patients for validation

**Fig. 5:**
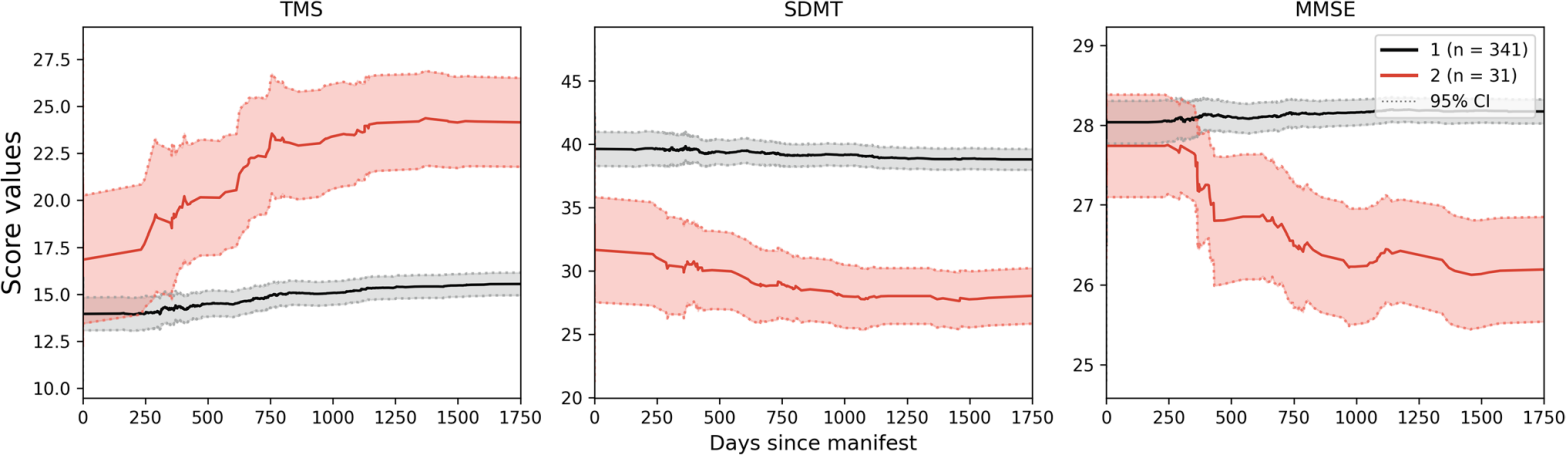
Mean progression cluster trajectories of pre-manifest patients. Applying the previously trained VaDER model on the multisymptom (TMS, SDMT, MMSE) trajectories of pre-manifest patients as a validation set results in two main trajectory clusters. Similar to the training scenario, Cluster 1 (black) contains the majority of patients (341), while Cluster 2 (red) only contains 31 patients. Again, the trajectories of first cluster show more stable patterns over time compared to the second cluster. Confidence intervals, especially for the second cluster, are larger than in the training case because of the significantly smaller number of patients

To further validate the generalization ability of the classification models, 372 pre-manifest at baseline patients were used as a further validation set. First, their conversion point from pre-manifest to manifest status was identified, as the visit at which the diagnostic confidence level altered from “motor abnormalities are likely signs of HD” to “motor abnormalities are unequivocal signs of HD”. Then, common disease time trajectories beyond this conversion time were clustered into two subtypes by applying the previously trained VaDER model. By only using the manifest part of the disease trajectory of an originally pre-manifest patient, we ensure the applicability of the model on this independent validation set, as it had been trained on manifest patients only. As can be seen in Fig. 5, Cluster 1 (black) contains, similar to the training set, the majority of patients, 341 in total, compared to only 31 patients in Cluster 2. Additionally, the trajectories of first cluster show more stable patterns over time in contrast to the second cluster. The larger confidence band of the second cluster in comparison to the training result, is due to the small number of patients within this group.

**Fig. 6:**
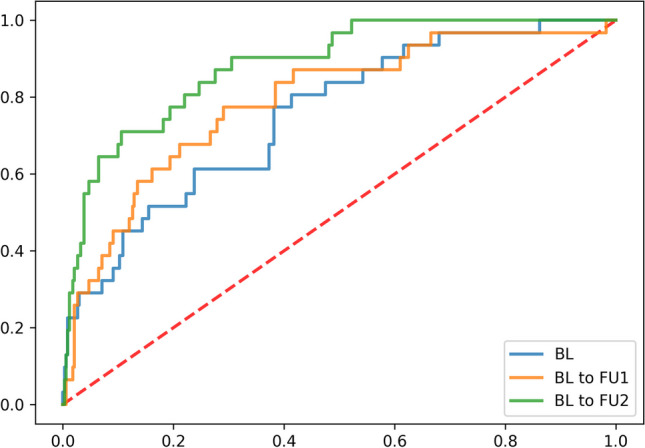
ROC for XGBoost classifiers applied on pre-manifest patients*.* The performance of the three RF classifiers BL only (blue), BLtoFU1 (orange), and BLtoFU2 (green) including different numbers of follow-up visits additional to the BL visit is presented. The area under the ROC curve (AUC) of the classifiers is 75%, 79%, and 88%, validating that classification performance increases with including additional visits

The assigned subtype of each patient was then used to validate the previously trained prediction models, BL, BLtoFU1, and BLtoFU2. ROC curves for the three different classifiers are shown in Fig. 6. The performance of the three different classifiers, BL only, BLtoFU1, and BLtoFU2, showed AUCs of 75%, 79%, and 88%. This demonstrated the generalization ability of the classification models and also strengthened the fact that classification performance increased with inclusion of additional follow-up visits, as expected.

Analysis of medication for five different indications showed no significance between the two subtypes (chorea: 0.1725, depression: $p=$ 0.8520, anxiety: $p=$ 0.1725, irritability: $p=$ 0.1987, cognitive decline: $p=$ 0.3048). Distributions of each medication in each subtype can be found in Supplementary Fig. S4.

## Discussion

The vision of a predictive, personalized, and preventive medicine demands to better tailor disease management and treatment to the needs of an individual patient by considering a large set of patient specific characteristics. Specifically, in the context of HD, there is an urgent need for a better personalized projection of symptom development to optimize counseling and planning of a patient’s future. In this regard, our work first identified two distinct HD subtypes within the Enroll-HD dataset, based on an AI-based clustering of patients’ disease trajectories that were aligned on a common disease timescale. From the two identified subtypes, surprisingly, one subtype relates to a large cluster showing a relatively stable disease progression, and the second, albeit smaller cluster, shows a dramatically more progressive disease trajectory. While this clustering was based on the UHDRS total motor score (TMS), UHDRS symbol digit modality test (SDMT), and the MMSE, the derived progressive subtype correlated with the CAG repeat length [49], underlining the robustness and validity of our clustering approach. In addition, the found subtypes could be replicated on an independent set of pre-manifest patients from the same cohort, further substantiating the validity of our approach.

Our approach is advantageous over other methods used and able to capture the heterogeneity in the disease course of HD, because integration of covariates into the modeling of each outcome allows to model effects of these covariates not only on disease stages but also on progression rate. Thus, we are able to adjust for potential covariate effects, such as age and gender on motor and cognitive symptoms. This approach also prevents bias in the data caused by disease diagnosis time, which is automatically considered when shifting patients to an earlier disease time on the common disease timescale, and thus, this strategy accounts for heterogeneity and uncertainty in the data. The subsequent use of VaDER for clustering the aligned disease trajectories allows then for simultaneous multivariate clustering rather than univariate clustering [30]. Furthermore, it allows for non-linear interactions across multiple scales for identification of the subtypes and not only captures a snapshot at a very specific time point in a patients’ medical history, but integrates the whole available disease course of each patient for specific clinical variables that are suggested for measuring the disease severity [30].

In addition to the above-mentioned differences in subtypes, further neurobehavioral, psychiatric, and cognitive scores were correlated with the found subtypes. In particular, the amount of cognitive impairment was the major difference between groups. Cognitive decline is correlated with the age at onset in HD patients [49]. Here, not only the scores used for clustering itself, namely, the MMSE and the SDMT, two already established scores reflecting morbidity and reduction in quality of life in HD, but also other cognitive scales, especially frontal-executive tests, as the letter verbal fluency, and Trail Making A and B, are distinguishing the subtypes. Thus, deficits in the overall cognitive performance, particularly the executive function, are associated with a drastically more progressive disease course, underlining previous data [50].

One potential confounder in our analysis may be the different intake of antichoreatic medication between groups. As our clustering is based on the TMS among others, intake of antichoreatic medication, leading to a lower TMS, may bias towards the slow progression group. However, a significantly higher amount within the fast progression cluster received antichoreatic medication. Thus, antichoreatic medication intake, likely reducing TMS motor scores, does not appear to influence clustering. Moreover, intake of antidepressive and anxiolytic medication was significantly more frequent in the fast-progressing group. These data suggest that the higher intake reflects the need to treat more advanced symptoms and does not cause a treatment bias in our clustering approach. In addition, the above-mentioned fact that cognitive scores are not used for clustering and CAG repeat length are among the strongest separating factors suggests a minor role of medication intake associated biases.

## Conclusion and expert recommendation in the framework of 3PM

The study identified two distinct HD progression subtypes, one relating to a larger cluster showing relatively stable disease progression and the second, smaller cluster, showing a more progressive disease trajectory. Characterization of the two subtypes showed a major difference in the amount of cognitive impairment between the groups. Multiple cognitive scales, especially frontal-executive tests, are distinguishing the subtypes. Thus, deficits in the overall cognitive performance are associated with a drastically more progressive disease course. Therefore, cognitive tests need to be taken into account, as they are more reflective of the disease progression than motor tests in many cases.

In relation to 3PM in HD, we see the contributions of our work as follows:

(i) *Predictive approach*: The subtype classification AI model can categorize HD patients based on their motor, as well as non-motor symptoms. Each individual patient can be assessed based on their personal profile and a clear and objectively measured disease progression is predicted. This offers HD patients a better perspective on their disease progression and allows them to organize their lives accordingly, which is the key to improving patient’s quality of life. In addition, a concrete prognosis could help doctors to optimize counseling and treatment of symptoms.
(ii) *Targeted prevention*: Cognitive deficits commonly appear in HD patients and a clear correlation with the disease progression is also known, but until now, they have been ignored in diagnosis, prevention, and prediction. This study clearly shows that non-motor symptoms, especially cognitive decline, are of major importance and need to be addressed in an optimized patient counseling and treatment (e.g., via cognitive training) by taking the results of cognitive test into account. But further work is needed for concrete advancements towards targeted prevention based on the two HD progression subtypes.
(iii) *Personalization of medical services*: A personalized projection of symptom development via the here-developed AI model helps to optimize counseling and thus the planning of each individual patient’s future. The individual prediction enables doctors to initiate the appropriate personalized therapies. With a reassessment at the next clinic visit and the resulting possible adjustment or refinement and concretization of the prognosis, the treatment and therapy can be adapted. Such refinements could improve the quality of patient’s life.

### Conclusion

In summary, we show that non-motor symptoms are of major importance for predicting and categorizing each patients’ disease progression pattern, even though they alone are not diagnostic. Our results substantiate a clinically well-known aspect of HD: the fact that cognitive decline is oftentimes more reflective of the progression of the disease than motor aspects. As a consequence, our analysis suggests that patient counseling should take into account results of the cognitive test found to be relevant in this study.

### Limitations and outlook

To further explore on our results, future work should focus on the correlation of our clustering with biological data such as MRI volumetry [51] or CSF biomarkers like neurofilament light protein [52]. This would help to delineate how far our clustering approach reflects underlying biological aspects of the disease and addresses the multimodal diagnostic concept to provide the maximum of clinically relevant information. In addition, neurobehavioral or psychiatric scores are currently underrepresented in our clustering approach, as well as in other studies [11, 46]. Yet, to include these scores in future work, we need more stable and easier to estimate non-linear mixed models allowing for the alignment of multivariate trajectories. Although, there is a multivariate version of the NLME available [33], estimating parameters of such a model is time-consuming and needs many manual steps, e.g., for finding or even tuning the start parameters for the estimation, which is inappropriate when integrating even more desired outcomes.

A further prospect regarding 3PM is to find the optimal treatment for each subtype. With the basis of our identified subtypes, clinical trial populations could now be adopted towards this vision. Enrichment of a clinical trial population with the rapid progressive subtype may help to show disease-modifying aspects of novel compounds, as this group is more likely to decline within typical trial periods of 1–2 years.

### Supplementary Information

Below is the link to the electronic supplementary material.

Supplementary file1 (DOCX 440 KB)

Supplementary file2 (XLSX 13 KB)

Supplementary file3 (XLSX 20 KB)

## Data Availability

The Enroll-HD database is available upon request from https://www.enroll-hd.org.

## Declarations

### Disclaimer

The funders played no role in the study design, data collection, analysis and interpretation of data, or the writing of this manuscript.
