# Supplementary material for: Unraveling progression subtypes in people with Huntington’s disease
Source: EPMA J. 2024 May 28;15(2):275–87. doi: 10.1007/s13167-024-00368-2 (PMC11148000; doi:10.1007/s13167-024-00368-2)
Supplement: Supplementary file 1 — Supplementary file1 (DOCX 440 KB) [file 13167_2024_368_MOESM1_ESM.docx]

Supplementary Information –

Unraveling progression subtypes in people with Huntington’s Disease

Tamara Raschka^†1^[
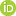
](https://orcid.org/0000-0003-2332-6137), Zexin Li^†1,2^, Heiko Gaßner^3,4^[
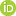
](https://orcid.org/0000-0003-2037-9460), Zacharias Kohl^5^, Jelena Jukic^3^, Franz Marxreiter^‡3,6^[
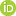
](https://orcid.org/0000-0002-5187-4344), Holger Fröhlich^‡1,2*^[
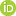
](https://orcid.org/0000-0002-5328-1243)

†,‡ Authors contributed equally

1. Department of Bioinformatics, Fraunhofer Institute for Algorithms and Scientific Computing (SCAI), Schloss Birlinghoven, 53757 Sankt Augustin, Germany
2. Bonn-Aachen International Center for IT, University of Bonn, Friedrich Hirzebruch-Allee 6, 53115 Bonn, Germany
3. Department of Molecular Neurology, University Hospital Erlangen, Friedrich-Alexander-Universität Erlangen-Nürnberg, 91054, Erlangen, Germany.
4. Fraunhofer IIS, Fraunhofer Institute for Integrated Circuits IIS, Am Wolfsmantel 33, 91058 Erlangen, Germany
5. Department of Neurology, University of Regensburg, Regensburg, Germany.
6. Center for Movement Disorders, Passauer Wolf, 93333 Bad Gögging, Germany.

*) corresponding author: [holger.froehlich@scai.fraunhofer.de](mailto:holger.froehlich@scai.fraunhofer.de)

Methods

## Dataset and patient selection criteria

### Definition of Pre-Manifest and Manifest Patients

Definition of pre-manifest and manifest patients rely on participant category defined in the “hdcat” variable of the dataset as described in the “Understand and Interpret the Data” document of Enroll-HD. Patients originally categorized as “pre-manifest/pre-motor-manifest HD” were assigned to the pre-manifest patient group and patients originally categorized as “manifest/motor-manifest HD” were assigned to the manifest patient group during this study.

### Non-Linear Mixed Effect Model

The non-linear mixed effect model used in this study follows the rational of [1,2]. Shortly, it is a non-linear mixed effect model with a generalized logistic function as the mean curve. More detailed, let $i$ be a subject with $m_{i}$ visits at time points $t_{i1}<\ldots<t_{im_{i}}$. $N$ number of subjects in total. The number of visits can be different across subjects, as well as the actual time points itself. Let $y_{ijk}$ be the observed value of outcome $k, k=1, \ldots, K$ of a specific subject $i$ at time point $t_{ij}$. $K$is the number of modelled outcomes. Then $y_{ijk}$ is defined as:

$$y_{ijk}=\mu_{k}\left( t_{ij}+x_{ij}^{T}\beta+z_{i} \right)+v_{ik}+\varepsilon_{ijk}, i=1,\ldots,N, j=1,\ldots,m_{i},$$

with $z_{i} \sim N\left( 0,\tau^{2} \right), v_{i}=\left( v_{i1},\ldots,v_{iK} \right) \sim N\left( 0,\Gamma\right),\varepsilon_{ij}\sim N\left( 0,diag\left( \sigma^{2} \right) \right), \sigma^{2}=\left( \sigma_{1}^{2}\ldots\sigma_{K}^{2} \right)$, where the function $\mu$ represents the shape of a mean progression curve. $x_{ij}^{T}\beta+z_{i}$ models time shift effects along the time axis (horizontal), with $x_{ij}^{T}\beta$describing the fixed effects as the difference in disease stages and $z_{i}$the random, unobserved variation. Vector $x_{ij}$contains the observations from the $p$potentially time-varying predictors of the $i$-th subject with $\beta$collating the associated beta coefficients. $v_{i}+\varepsilon_{ij}$ models the vertical random effects, where $v_{i}$ describes the time-invariant shift between subjects that are normally distributed with$\Gamma$ as an unknown covariance matrix modelling the vertical correlation across time-invariant deviations in scores, and $\varepsilon_{ij}$ the measurement error. $\varepsilon$ is here identically distributed and independent of all other effects. The random effects, $z_{i}$ and $v_{ik}$, which model the shift in disease stages and the variation in clinical scores across different samples, are independent. Notably, the latent time scale generated by $t_{ij}+x_{ij}^{T}\beta+z_{i}$ forms a global timescale for disease progression in the study population and is therefore, the only term that is not dependent on $k$. More details can be found in [1,2].

During the here described analysis the mean curve is formulated as a generalized logistic function:

$$\mu\left( t \right)=A+\frac{K-A}{\left( 1+e^{\left( -B\left( t+s \right) \right)^{v}} \right)}+c$$

Where $A$ is the left and $K$the right asymptotic value which reflects the minimal and maximal possible value of a specific clinical measure. Parameters $B$ and $v$ define the curvature of the function, with $B$ as time scaling parameter and $v$ as an asymmetry parameter. A shift in time is modelled by $s$ as horizontal shift and a vertical shift can be modelled by $c$.

During the modelling the values of $A$ and $K$ are fixed according to the actual modelled clinical score. A fixed effect of the baseline manifestation status of the patients on the time shift is included, such that the parameter estimates of s describes the differences between the pre-manifest and manifest patient group over the continuous latent time scale. This difference is modelled relative to the pre-manifest patient group, meaning that $t=0$ on the latent time scale corresponds to the average status of the pre-manifest patients at baseline. Parameters $B$ and $v$ need a start value definition within the *progmod* procedure. These start values were optimized with a grid search as a hyperparameter optimization. Based on the log-likelihood we selected the best model with the starting parameters shown in **Table S1**.

**Table S1 Hyperparameter list for NLME model**

| **Effect type** | **Parameter** | **Value** |
| --- | --- | --- |
| fixed | A – motor score | 0 |
|  | A – sdmt1 | 110 |
|  | A – MMSE | 30 |
|  | K – motor score | 124 |
|  | K – sdmt1 | 0 |
|  | K – MMSE | 0 |
|  | B – motor score | 0.005 |
|  | B – sdmt1 | 0.037 |
|  | B – MMSE | 0.007 |
|  | v – motor score | 1.3 |
|  | v – sdmt1 | 1.2 |
|  | v – MMSE | 1.8 |
|  | c | 0 |
| random | s – intercept | -200 |
|  | s – manifest | 50 |

### Multivariate clustering of clinical trajectories

A clustering of clinical trajectories was performed using the previously published VaDER approach [3]. Following a long short-term memory (LSTM) network and recurrent variational autoencoder, VaDER learns a low-dimensional representation of the data as Gaussian mixture model. The mixture components can then be used for a probabilistic cluster assignment of patients.

A hyperparameter optimization was performed with a grid search on a set of hyperparameters including the number of clusters, the number of units per hidden layer, the learning rate, and the batch size. The best parameter set was selected based on the best model performance, which was measured here with the help of the prediction strength of each model against a null model build on a random sampling of the dataset [4]. A valid hyperparameter set led to a significant difference in the prediction strength between both models. The number of clusters was defined as the smallest number of clusters leading to that significance (see **Figure S1**).


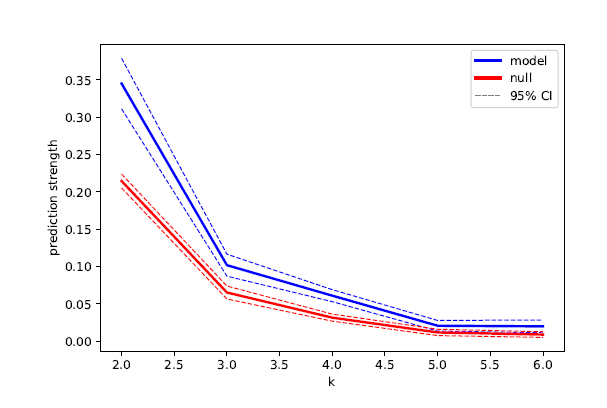


**Figure S1: Prediction strength for best hyperparameter setting.**Prediction strength is plotted for VaDER versus a null model based on random permutation of cluster memberships for several choices of the number of clusters k.

The final model was trained with the optimal hyperparameter set (**Table S2**), including the optimal number of clusters. Final cluster assignment was based on the consensus of 20 independent runs of the clustering.

**Table S2: Hyperparameter list for VaDER**

| **Parameter** | **Value** |
| --- | --- |
| Number of hidden layers | 2 |
| Number of units first layer | 128 |
| Number of units second layer | 16 |
| Learning rate | 0.00001 |
| Batch size | 32 |
| Alpha | 1 |
| k (number of clusters) | 2 |

### Machine learning classifiers

For training Random Forest and XG Boost classifiers on baseline data only (BL), as well as baseline and follow-up data (BLtoFU1 and BLtoFU2), hyperparameter optimization was performed with random search with 100 parameter settings from the hyperparameter space shown in **Table S4** for Random Forest and in **Table S5** for XG Boost.

**Table S4: Random Forest classifier hyperparameter space**

| **Parameter** | **Grid values** |
| --- | --- |
|  |  |
| Max. tree depth | [None, 10, 20, 30, 40, 50] |
| Min. samples split | [0.05, 0.1, 0.15, 0.2, 0.25] |
| Min. samples leaf | [10, 20, 50, 100, 200] |
| ccp alpha | [0.001, 0.01, 0.1] |

**Table S5: XG Boost classifier hyperparameter space and optimal values**

| **Parameter** | **Grid values** | **Optimal value** | | |
| --- | --- | --- | --- | --- |
|  |  | **BL** | **BLtoFU1** | **BLtoFU2** |
| Number of estimators | [100, 200, 300, 400, 500] | 500 | 500 | 500 |
| Max. tree depth | [3, 5, 7, 9] | 3 | 5 | 5 |
| Min. child weight | [1, 3, 5] | 3 | 3 | 5 |
| Gamma | [0.0, 0.1, 0.2, 0.3, 0.4] | 0.2 | 0.0 | 0.2 |
| Reg lambda | [0.0, 0.1, 0.2, 0.3, 0.4, 0.5, 0.6, 0.7, 0.8, 0.9, 1.0] | 0.3 | 0.8 | 1.0 |
| Reg alpha | [0.0, 0.1, 0.2, 0.3, 0.4, 0.5, 0.6, 0.7, 0.8, 0.9, 1.0] | 0.5 | 0.7 | 0.9 |
| Subsample | [0.6, 0.7, 0.8, 0.9] | 0.7 | 0.6 | 0.7 |
| Colsample by tree | [0.6, 0.7, 0.8, 0.9] | 0.7 | 0.9 | 0.9 |

10-fold nested cross validation results of both Random Forest and XG Boost are listed in **Table S6**, showing that XG Boost algorithm is outperforming Random Forest method. Thus, XG Boost was used as the method of choice to train the final models. Optimal hyperparameters were here found within a 10-fold nested cross validation procedure based on the best AU-ROC. The optimal value for each hyperparameter in each of the three learned final XG Boost models is shown in **Table S5**.

**Table S6: Average AU-ROC from nested cross-validation of Random Forest and XG Boost models**

| **Random Forest** | | | **XG Boost** | | |
| --- | --- | --- | --- | --- | --- |
| **BL** | **BLtoFU1** | **BLtoFU2** | **BL** | **BLtoFU1** | **BLtoFU2** |
| 0.87 ± 0.02 | 0.89 ± 0.02 | 0.92 ± 0.02 | 0.95 ± 0.01 | 0.99 ± 0.00 | 0.99 ± 0.00 |

Results

Clinical characterization of progression sub-types

Statistical tests regarding the differences in the features, aggregated in the top 10 most important features for the prediction of progression subtypes found via SHAP analysis, were conducted. The distributions for the TOP5 features of each classifier (BL, BLtoFU1, BLtoFU2) are shown in **Figure S2**.
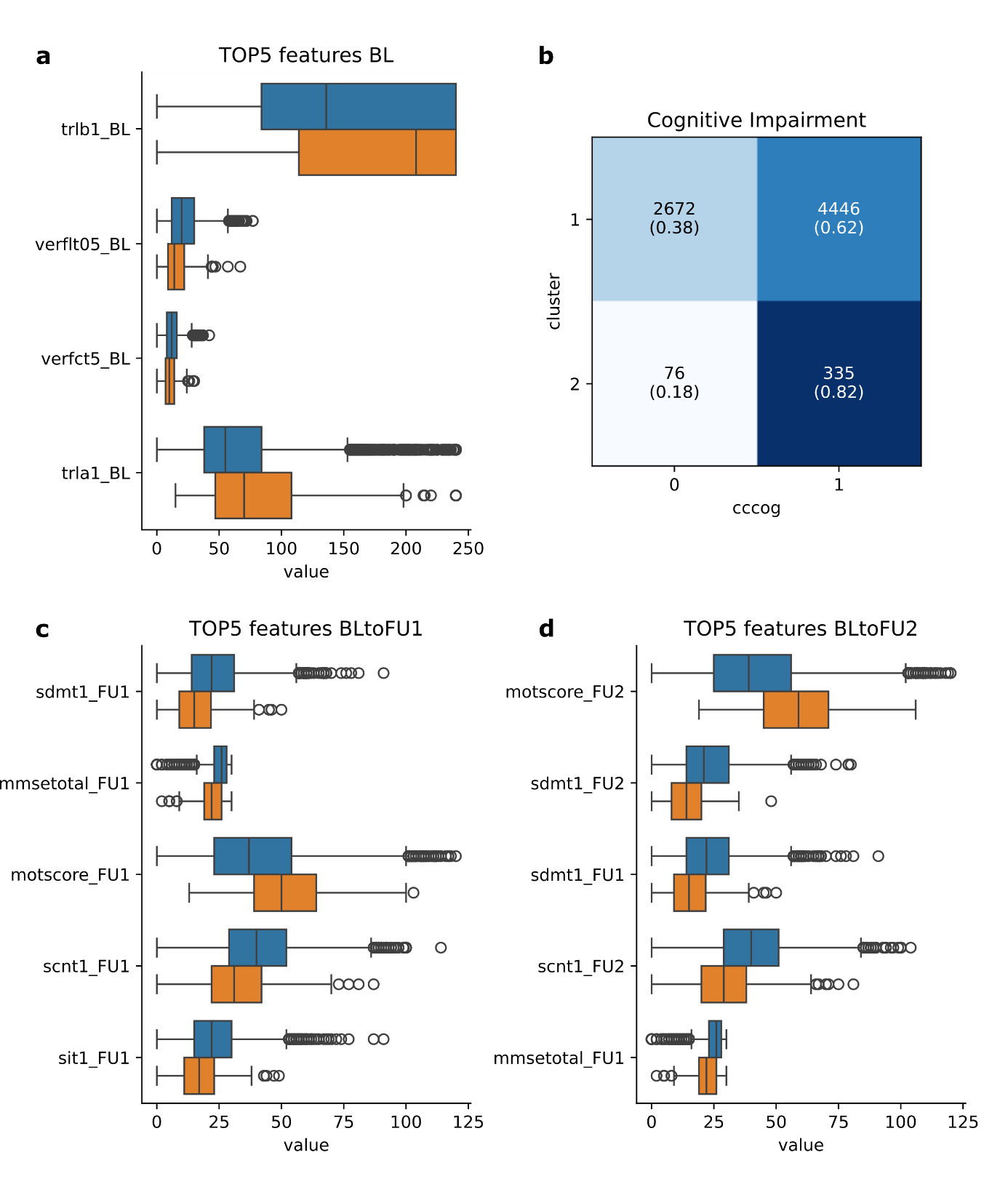


**Figure S2: Distribution of TOP5 significantly different** **features.**
Shown are the distributions of the TOP5 significantly different features from the BL (A + B), BLtoFU1 (C), and BLtoFU2 (D) classifiers, where subtype 1 is coloured in blue, and subtype 2 in orange.

In addition, the medication taken by patients within the subtypes were tested and their distributions can be found in **Figure S3**.


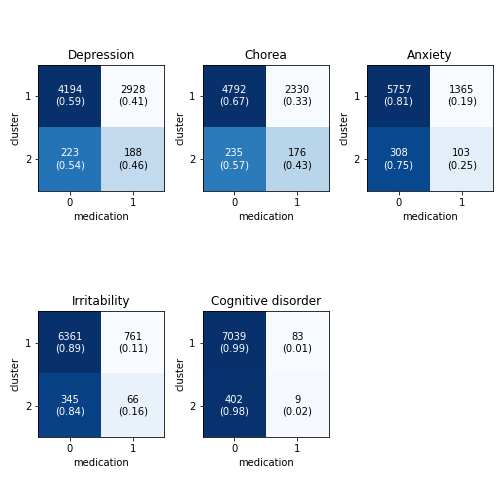


**Figure S3** **Medication indication across subtypes**Shown are the distributions of the indications for medication intake in the two subtypes, where medication = 0 reflects no medication intake and medication = 1 an intake for this indication.

## Application of model on pre-manifest patients for validation

The medication taken by patients from the validation dataset within the subtypes were tested and their distributions can be found in **Figure S4**.


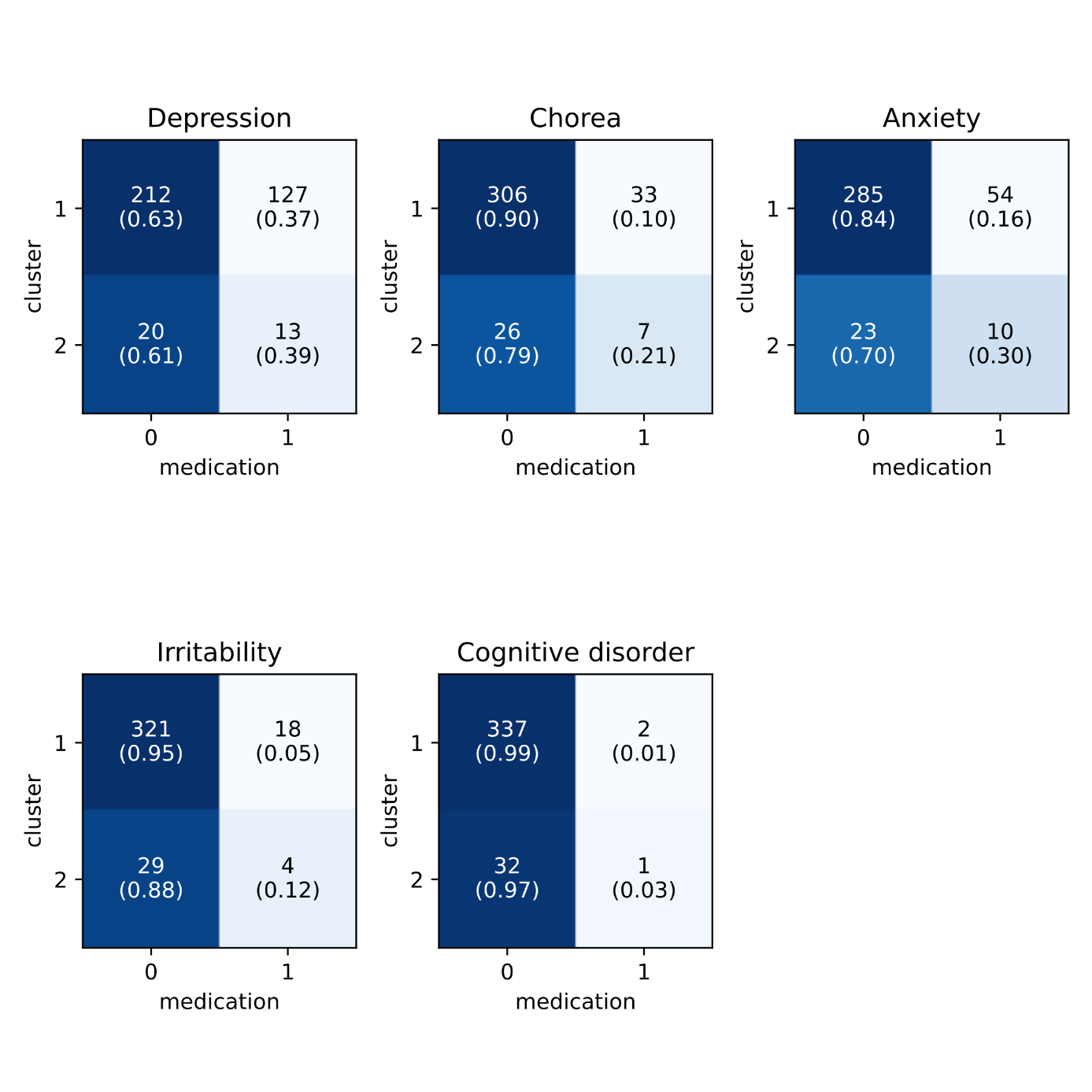


**Figure S4 Medication indication across subtypes of validation patients**Shown are the distributions of the indications for medication intake in the two subtypes for patients from the validation dataset, where medication = 0 reflects no medication intake and medication = 1 an intake for this indication.

References

1. Raket, L. L. Statistical Disease Progression Modeling in Alzheimer Disease. *Front. Big Data* **3,** 24 (2020). https://doi.org/10.3389/fdata.2020.00024

2. Kühnel, L., Berger, A.-K., Markussen, B. & Raket, L. L. Simultaneous modeling of Alzheimer’s disease progression via multiple cognitive scales. *Stat. Med.* **40,** 3251–3266 (2021). https://doi.org/10.1002/sim.8932

3. de Jong, J. *et al.* Deep learning for clustering of multivariate clinical patient trajectories with missing values. *GigaScience* **8,** giz134 (2019). https://doi.org/10.1093/gigascience/giz134

4. Tibshirani, R. & Walther, G. Cluster Validation by Prediction Strength. *J. Comput. Graph. Stat.* **14,** 511–528 (2005). https://doi.org/10.1198/106186005X59243
